# Biosecurity assessment and seroprevalence of relevant poultry diseases in Saint Kitts commercial poultry premises

**DOI:** 10.1007/s11250-026-05099-2

**Published:** 2026-05-28

**Authors:** Stephanie R. Benedict, Rodrigo A. Gallardo, Brendan Liebross, Marcus Machado, Luis Pablo Hervé-Claude

**Affiliations:** 1 Ross University School of Veterinary Medicine, Basseterre, St. Kitts and Nevis; 2 School of Veterinary Medicine, University of California, 1089 Veterinary Medicine Dr., Davis, CA 95616 USA; 3 Lewyt College of Veterinary Medicine, Long Island University, 720 Northern Blvd, Brookville, NY 11548 USA; 4 Animal Diagnostic Laboratory, Department of Veterinary and Biomedical Sciences, College of Agricultural Sciences, Pennsylvania State University, 131 Pastureview Rd, University Park, PA 16802 USA

**Keywords:** Saint Kitts & Nevis, Commercial flock, Seroprevalence, Caribbean, Poultry

## Abstract

Saint Kitts (St. Kitts) and Nevis is a federation of two islands located within the West Indies. St. Kitts is anecdotally known to have commercial egg production, but disease prevalence and biosecurity practices of these farms have not previously been studied. In this study, we evaluated biosecurity practices of 11 commercial farms through a biosecurity questionnaire given to the farmers and collected blood and oropharyngeal swab samples from birds at each farm to evaluate disease prevalence. All surveyed egg-layer farms had deficiencies in conceptual, structural, and procedural biosecurity. Favorably, most workers used dedicated farm shoes and clothes and limited visitors. The inadequate cleaning of houses, handling of mortality, and multi-age facilities raised concerns for disease transmission. For seroprevalence, antibodies against avian influenza virus (AIV), Newcastle disease virus (NDV), infectious bronchitis virus (IBV), and *Salmonella enterica* subspecies *enterica* serovar Enteritidis (SE) were detected using ELISA kits. Most farms had positive antibodies to IBV (90.9%, 10/11), AIV (81.8%, 9/11), and all farms were positive for SE and NDV (100%, 11/11). Molecular testing for NDV and AIV antigen detection were negative. Chicks imported from hatcheries in other countries received vaccinations for IBV and NDV, suggesting that chicks are transmitting vaccinal strains when integrated into multi-age layer houses. SE seroprevalence in all farms, paired with poor nest availability, egg sanitation practices, and ineffective house cleaning raise food safety concerns. The results of this study highlight the need for disease prevention programs in St. Kitts commercial poultry production, to safeguard both animal and human health.

**Supplementary Information:**

The online version contains supplementary material available at 10.1007/s11250-026-05099-2.

## Introduction

Poultry production is an important source of protein for most countries (OECD/FAO 2025) and countries within the Caribbean are no exception. The Federation of Saint Kitts (St. Kitts) and Nevis consist of two small islands located in the Eastern Caribbean. The country’s agricultural production has significantly varied over the years (Department of Statistics, Ministry of Sustainable Development 2022), with food imports costing annually $140 million (St. Kitts Nevis Information Service 2024a), from those nearly $20 million were chicken products in 2022 (St. Kitts Nevis Information Service 2023). Despite this, there is an incipient poultry industry on the island, anecdotally known to supply table eggs to local grocery stores. With the country’s stance on increasing poultry production, it is important to understand local disease challenges and establish preventative programs.

In low- and middle-income countries, poultry mortality from viral infections contribute to 24.5% of economic losses per production cycle in backyard poultry farming (Muñoz-Gómez et al. 2025). Diseases, like highly pathogenic avian influenza (HPAI) and Newcastle disease can result in widespread financial loss due to mass mortality events. Beyond mortality events, infectious bronchitis virus (IBV), lentogenic NDV and low pathogenic avian influenza (LPAI) can contribute to respiratory disease and egg production losses, negatively impacting economics in the layer industry (Dane Lester Hartley et al. 2021; Gainor and Ghosh 2022; Reid et al. 2023).The Caribbean is no exception to these challenges. The Cayman Islands had its first HPAI outbreak in 2026, which resulted in the loss of 65 domestic birds after exposure to an infected wild duck (OIE-WAHIS 2026). In addition, Newcastle disease virus (NDV), has been detected in St. Kitts and reported to the World Organization for Animal Health back in 1971 (Gainor and Ghosh 2022).

While the financial impact of these diseases is not well described in the region, these losses have been characterized in other countries. As an example, the 2022 United States HPAI outbreak has exceeded $1.4 billion USD in related costs by November 2024 and is still ongoing (Animal and Plant Health Inspection Service, USDA 2024). Additionally, NDV infected layer flocks in Iran resulted in a loss of 5,269 billion Iranian Rial from egg production losses (Charkhkar et al. 2024) and IBV in Brazil resulted in US$4,210.8 total loss per 1,000 birds in 42-week-old breeders (Colvero et al. 2015).

Similarly, foodborne disease caused by non-typhoid *Salmonella* spp., most notably *Salmonella enterica* subspecies *enterica* serovar Enteritidis (SE), is costly from a productive perspective and in poultry products is a food safety concern. The regional annual cost associated with gastrointestinal illness in humans was US$389,000 and US$19,736,340 in St. Lucia and Trinidad and Tobago, respectively (Donna Morrison et al. 2022). A review of foodborne diseases in Central America and the Caribbean noted *Salmonella* spp. as the most prevalent foodborne pathogen, being 38% of reported cases, and was primarily linked to the consumption of undercooked poultry and raw eggs (Severino et al. 2025).

Regionally, studies to understand poultry disease challenges have occurred in different Caribbean nations (Adesiyun et al. 2014; Brown Jordan et al. 2018; Dane Lester Hartley et al. 2021), but, to date, there are no published studies on biosecurity nor disease prevalence in St. Kitts poultry farms. A previous study evaluated pathology and seroprevalence in free roaming chickens and found evidence of exposure to multiple poultry pathogens of commercial importance (i.e., NDV and IBV) (Bolfa et al. 2019), highlighting the need to evaluate biosecurity and serological prevalence of poultry diseases in commercial premises.

Biosecurity surveys have been widely employed in both backyard and commercial poultry research to easily obtain information on disease mitigation practices utilized and aid risk assessments (Brochu et al. 2021; Gemeda et al. 2026). In this study, a questionnaire was utilized to evaluate aspects of conceptual, structural, and operational biosecurity practices. For seroprevalence, antibodies against NDV, AIV, IBV, and SE were assessed. Additionally, limited PCR assessments of choanal swabs were performed to identify viral particle presence in healthy birds. The goal was to evaluate poultry diseases, food safety relevance, and better understand challenges in the island’s poultry industry.

## Materials and methods

### Poultry flocks

**Fig. 1:**
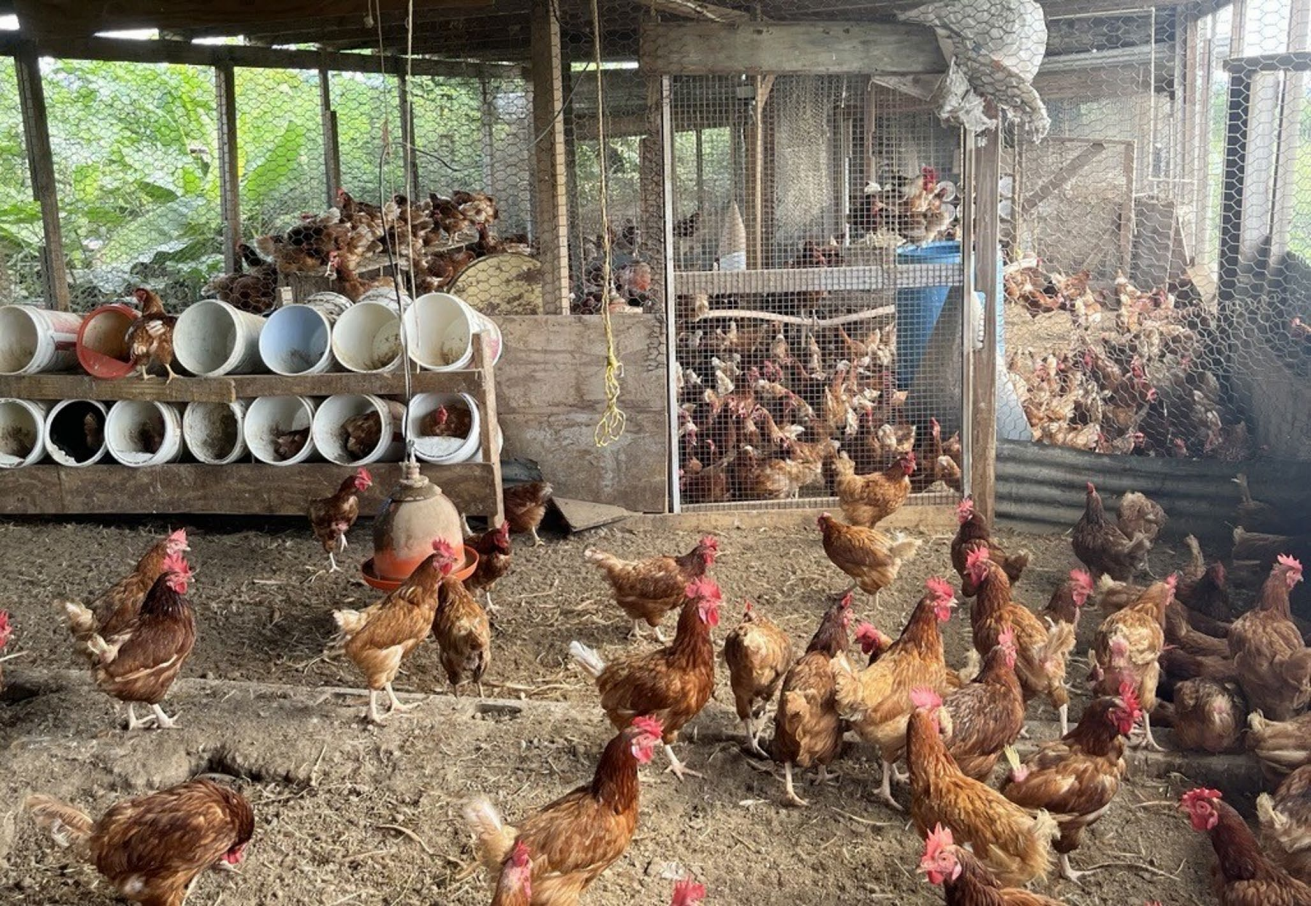
A view of an exemplary St. Kitts poultry farm in 2023. Note the infrastructure and the soil floor

Commercial poultry farms were sourced through the Ministry of Agriculture, Fisheries and Marine Resources of St. Kitts and Nevis. No poultry farmers census data was available in St. Kitts at the time of this study, but the number of farms was estimated to be 20 semi-commercial farms including one larger facility. In the face of this extremely small sampling frame, it was decided to attempt to collect data from all possible farms. Sample size calculation was not required based on the low number of farms and the complete absence on disease prevalence and farm biosecurity information to base a sample size calculation. After cross verification of active registered poultry farms, 10 farmers were contacted by the Ministry, and one additional farmer was contacted after being referred to by one of the visited farmers. While farms had minor structural differences, an exemplary flock is shown in Fig. 1 (Fig. 1). An informed consent form was signed by each farmer prior to sampling and data collection. This project was performed under research authorization granted by Ross University School of Veterinary Medicine (RUSVM), IACUC#: 22.10.05.

### Biosecurity survey

A biosecurity questionnaire was adapted from a previous study (Derksen et al. 2017). The goal was to evaluate demographics, biosecurity, and sanitation in the commercial poultry farms visited. Seven categories were created: poultry housing, wildlife and domestic animal interactions, cleaning and disinfection protocols, flock biosecurity and health, farm layout, value chain, and egg food safety. The full survey is provided as a supplementary file (Online Resource).

### Sample collection

Sample size was calculated based on the reported IBV seroprevalence found in wild chicken on island (Bolfa et al. 2019), using a simple proportion (apparent prevalence) with a precision of 0.05 and a confidence level of 0.95. This resulted in a sample size of 205 samples needed across all farms. Blood was collected from the ulnar vein of 20 birds per farm; sera was extracted to be used in ELISA tests to detect the presence of antibodies against 4 relevant poultry diseases. A total of 220 sera samples were collected from the 11 tested farms. Oropharyngeal swabs were collected from 10 birds in two pools of 5 swabs for a total of 22 pools (two pools per farm).

### Serological tests

Coagulated blood samples were chilled in the refrigerator prior to centrifugation at 3000 rpm for 5 min. Serum was extracted and transferred into a fresh 1 mL Eppendorf tube. Samples were then frozen in -20 °C until all 11 farms were sampled. Antibody titer determination was performed using commercial ELISA kits for AIV, NDV, IBV, and SE (IDEXX, Westbrook, ME) following manufacturers’ instructions. Optical density was measured using a spectrophotometer (BioTek instruments, Winooski, VT).

### Molecular antigen detection

If birds tested positive for AIV or NDV antibodies by ELISA, Finders Technology Associates (FTA) cards impressed with oropharyngeal swab pools obtained in those farms were tested for the presence of the antigen using a reverse transcriptase quantitative polymerase reaction (RT-qPCR). RNA was extracted from FTA cards using a King Fischer automatic extractor and Mag Max viral RNA extraction kits (Thermo Fischer, USA). Viral RNA was used in an RT-qPCR to detect the presence of AIV and/or NDV as described in (Spackman et al. 2002; Wise et al. 2004).

### Statistical analysis

ELISA S/P ratios for the four assessed diseases were recorded, as to provide a further view of titer distribution within and among farms per pathogen. R statistical software was used for all analyses. After graphical and statistical evaluation using Shapiro-Wilk test determined the data to be non-normally distributed, non-parametric Kruskal-Wallis test was used for further statistical comparison to indicate any statistically significant differences for each pathogen (*p* < 0.05). Post-hoc analysis to compare significant differences between farms for each pathogen was done using Dunn’s test.

## Results

### Biosecurity and sanitation survey

Eleven egg layer farms were visited and surveyed. At the time of the study, the farmers interviewed reported a total of 21,024 laying hens with an overall daily production of 14,336 eggs (roughly 68.2% daily egg production). The mean flock size was 1,911 hens (range 24 − 12,000) with a median of 1,000 hens. The mean and median egg production per farm was 1533.6 and 540 eggs per day, respectively.

Most of the farms surveyed (45.5%, 5/11) reported the nearest public road to be more than 91.4 m (100 yards) away, with only two (18.2%, 2/11) reporting it to be less than 45.7 m (50 yards). Seven (63.4%, 7/11) farms reported the nearest poultry farm to be less than 365.8 m (400 yards) away. Furthermore, five farmers (45.5%, 5/11) mentioned that the nearest poultry farms were in the same neighborhood. Freshwater bodies are mostly absent from the island, but one farmer (9.1%, 1/11) reported seeing waterfowl in the vicinity of the farm. Regarding wild birds, (45.5%, 5/11) farmers see them with varying frequency. Seven out of 11 farmers (63.6%) claimed to be able to keep wild birds away from their flock, with 18.2% (2/11) not seeing them on the premises. No farm reported having vermin control plans.

For infrastructure, most of the farms reported perimetral fencing (81.8%, 9/11). Nine of the farms had cage free systems (81.8%, 9/11), with one having outdoor access. The rest (18.2%, 2/11) had battery cages. Footbaths were absent from all farms, but seven farmers (63.6%, 7/11) reported to use dedicated shoes and clothes. Most farmers (63.6%, 7/11) reported not having protected feeders with feed spills not immediately cleaned. No farmer shared equipment with other farms. The visit regime ranged from no visitors allowed on seven farms (63.6%, 7/11) to unrestricted visits in three of them (27.3%, 3/11).

For cleaning and disinfection, the frequency of cleaning was found to be dissimilar between farms, with some reporting “weekly” to “every three months” or “when needed”. Most farmers (72.7%, 8/11) indicated that the cleaning process included equipment servicing and removal of superficial soil. The remainder (27.3%, 3/11) only removed litter. The litter and bedding material were mainly used as fertilizers either on their own or on neighboring farms. Seven farmers claimed to use disinfectants (63.6%, 7/11). Some of the products categorized as disinfectants were chlorine, vinegar, zeolites, quaternary ammonia, and “black disinfectant” – a regionally commercialized natural tar-based formulation.

All farmers inspected their flock daily while record keeping was sparse with only three (27.3%, 3/11) recording egg production, feed consumption, and mortality. Consequently, (72.2%, 8/11) farmers claimed to have no records. When records were available, these were either on loose paper or notebooks. Therefore, no reliable information could be obtained on hens age, as no records were available and groups of hens were mostly mixed. All but one farm offered adult laying hens for sampling. The remaining farm was newly established and received pullets of unreported age from another producer on island. Ten farmers (90.9%, 10/11) imported chicks from the U.S. as replacements, with only one farmer (9.1%, 1/11) sourcing his birds locally from neighbors. Only three farms culled old hens (27.3%, 3/11) while the others sold old hens on site (72.2%, 8/11), gave them away (54.5%, 6/11), sold them to other farms (9.1%, 1/11), or kept them as pets (9.1%, 1/11).

Mortality was handled differently between farms, (54.5%, 6/11) buried dead birds, (27.3%, 3/11) disposed of them as garbage, and (18.2%, 2/11) distributed dead birds to people or animals as food/feed, or as bait to keep monkeys away. Occasionally, one of the farms sent dead birds to Ross University for pathology examination and student training. All farms claimed informal quarantine procedures isolating new birds on arrival. No bird movement was reported, as there seems to be no live bird markets nor bird shows on the island. No farm had a written biosecurity protocol.

A large variety and number of nest types were identified across farms, including wooden boxes, plastic containers, complete or half tires, and other types of structures and objects. The nest-to-hen ratio was widely variable, with most houses having evidently few nests, ranging from 1:1 to 1:52 individual nest per hen.

Egg collection and in-farm processing were different in all farms. Eggs were washed on most farms. The non-standardized procedure observed was as follows: eggs are collected once a day by hand and stored in buckets. Eggs are then submerged in large numbers in a bucket with water, sometimes with a cleaning agent (dish detergent, chlorine or vinegar). One-by-one these eggs are extracted from the bucket and brushed with a wipe or cloth (72.27%, 8/11) that was changed on a weekly basis or used until damaged. One farmer (9.1%, 1/11) claimed to wash the wipe/cloth every week. Afterwards, eggs are rinsed in another bucket with water and stored in another container where they dry (usually a bucket with holes). This procedure is performed directly outside the poultry houses.

### Serology results

ELISA results confirmed antibody presence in commercial flocks. A summary of the results can be seen in Table 1. All but one farm showed positive IBV specific antibodies (90.9%, 10/11). All but three farms were positive for AIV specific antibodies (72.7%, 8/11), and all 11 farms were positive for NDV and SE specific antibodies. Most farms shared all four positive antibody results (63.6%, 7/11).

**Table 1:** ELISA serological results from eleven commercial egg laying farms in St. Kitts, St. Kitts & Nevis, West Indies, 2022–2023

Farm ID	Newcastle Disease Virus		Avian Influenza Virus		Infectious Bronchitis Virus		Salmonella Enteritidis	
	% positive	Status	% positive	Status	% positive	Status	% positive	Status
1	50% (10/20)	(+)	10% (2/20)	(+)	95% (19/20)	(+)	55% (11/20)	(+)
2	70% (14/20)	(+)	10% (2/20)	(+)	100% (20/20)	(+)	40% (8/20)	(+)
3	50% (10/20)	(+)	5% (1/20)	(+)	100% (20/20)	(+)	60% (12/20)	(+)
4	50% (10/20)	(+)	5% (1/20)	(+)	100% (20/20)	(-)	40% (8/20)	(+)
5	60% (12/20)	(+)	10% (2/20)	(+)	100% (20/20)	(+)	40% (8/20)	(+)
6	75% (15/20)	(+)	15% (3/20)	(+)	100% (20/20)	(+)	85% (17/20)	(+)
7	75% (15/20)	(+)	15% (3/20)	(+)	100% (20/20)	(+)	95% (19/20)	(+)
8	55% (11/20)	(+)	0% (0/20)	(-)	100% (20/20)	(+)	85% (17/20)	(+)
9	30% (6/20)	(+)	0% (0/20)	(-)	0% (0/20)	(+)	20% (4/20)	(+)
10	80% (16/20)	(+)	5% (1/20)	(+)	100% (20/20)	(+)	65% (13/20)	(+)
11	95% (19/20)	(+)	0% (0/20)	(-)	85% (17/20)	(+)	45% (9/20)	(+)
**Average**	**62.7%**	**11/11 (+)**	**6.8%**	**8/11 (+)**	**89.1%**	**10/11 (+)**	**57.3%**	**11/11 (+)**

**Fig. 2:**
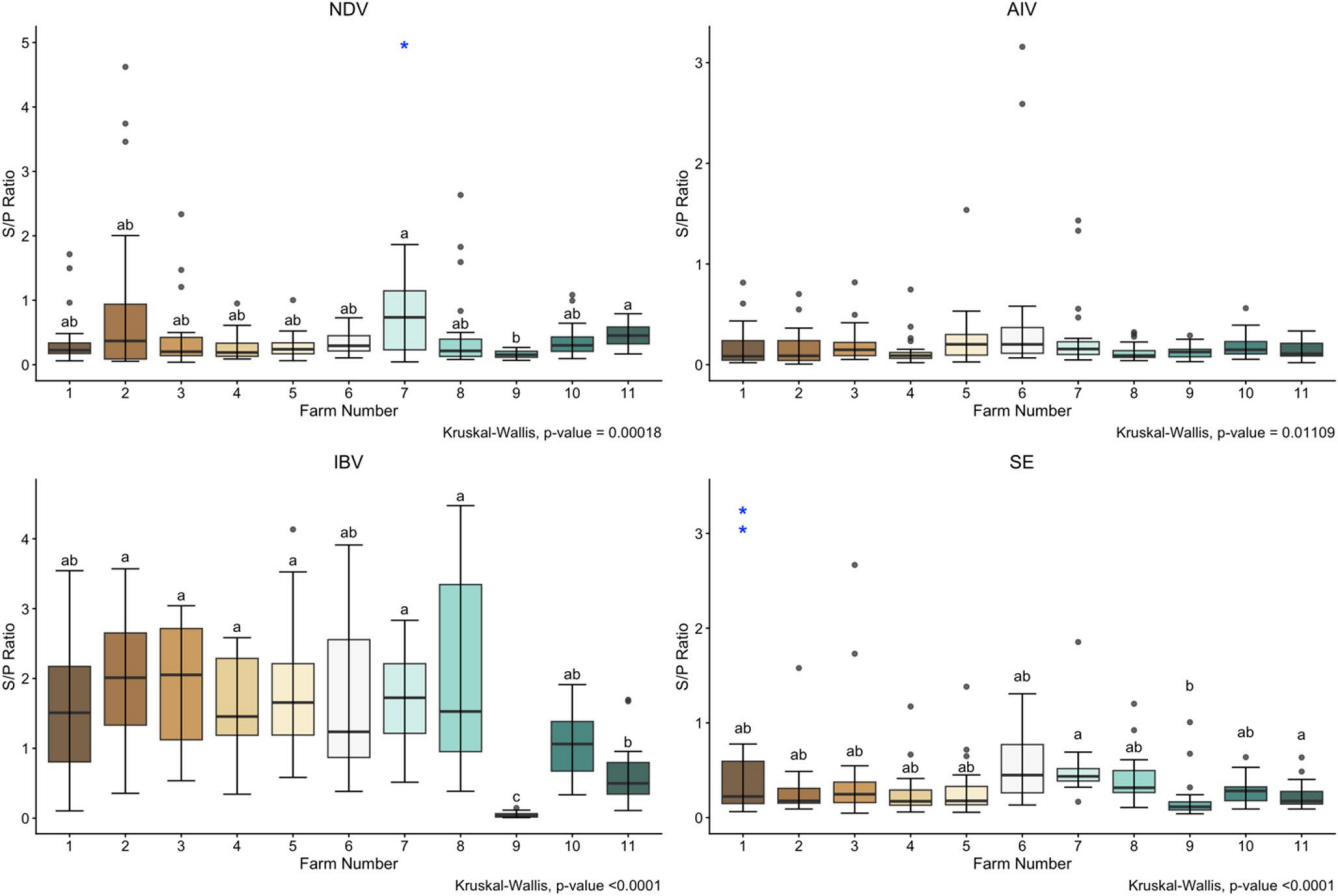
Distribution of S/P ratios for each pathogen per farm. Boxplots represent interquartile ranges, medians, and outliers. Statistically significant differences between farms were found for each pathogen. The p-values according to the Kruskal-Wallis test are displayed in the bottom right corner of each graph. Different letters displayed above the boxplots indicate significant differences between farms (*p* < 0.05) for NDV, IBV, and SE. Outliers that were cut off for better visualization of boxplots are depicted with the * symbol in NDV farm 7 and SE farm 1

Boxplots were created to show the distribution of S/P ratios for all four studied diseases per farm (Fig. 2). Kruskal-Wallis test for each pathogen indicated statistically significant differences between farms for all for studied disease antibodies (NDV *p* = 0.00018; AIV *p* = 0.01109; IBV *p* < 0.0001; SE *p* < 0.0001). Dunn’s test determined multiple statistical differences (*p* < 0.05) between farms for NDV, IBV, and SE. NDV seroprevalence differed between farms 7 vs. 9 and farms 9 vs. 11. IBV seroprevalence differed between farm 9 vs. farms 1–8 and 10; Farm 11 vs. farms 2–5, 7–8. SE seroprevalence differed between farms 4 vs. 6; farm 7 vs. farm 2, 4, 5, 9 and 11; and farm 9 vs. 6, 7, and 8. Despite the statistically significant difference determined by the Kruskal-Wallis test, the Dunn’s test showed no differences between farms for AIV serology.

### Antigen detection

Oropharyngeal swabs obtained from farms with positive ELISA results to NDV and AIV proved to be negative to antigen detection by RT-qPCR.

## Discussion

Biosecurity is considered one of the most important preventative methods to protect flocks from infectious diseases (Julianingsih et al. 2025; Kovács et al. 2025). All the surveyed egg-layer farms lacked or had deficiencies in areas of conceptual, structural, and procedural biosecurity. Despite this, most workers used dedicated shoes and clothes when working on the premises and limited visitors. This systematic lack of biosecurity is not unique for the Caribbean, with many regions of the world having small, semi-commercial producers selling eggs for consumption without proper biosecurity or inspection (Batista et al. 2020; Brochu et al. 2021; Di Francesco et al. 2025).

At the time of this survey, 90.9% of commercial layers in St. Kitts were sourced from hatcheries in Miami and Barbados where they were vaccinated for various pathogens prior to export. No vaccines were available in St. Kitts for further vaccinations. Imported chicks were introduced into multiple age laying houses that served as brooders and production units. Producers reported keeping hens for years, typically until they died naturally. Multi-age flocks are a known risk factor for pathogen circulation due to constant new bird introductions (Patch et al. 2025) and their inability to provide downtime to reduce pathogen load (Gingerich 2017). Most St. Kitts farms also had dirt floors, making proper disinfection impossible due to the presence of organic material.

Egg handling was also deficient, as eggs were washed and stored without refrigeration, thus increasing the food safety risk for this community. The mean annual temperature in St. Kitts and Nevis was 80.6 °F (27 °C) (St. Kitts and Nevis Ministry of Environment 2023). This increases potential bacterial growth if eggs are washed, losing their protective cuticle (Jones et al. 2018; Li et al. 2025). In countries where eggs are washed, storing temperature should be 45 °F (7.2 °C) (Food Safety and Inspection Service, USDA 2020). Many countries in Europe and Latin America prohibit commercial egg washing (European Commission 2023). The idea is to preserve the egg cuticle that acts as a barrier for bacterial colonization in absence of refrigeration. Therefore, the current egg sanitation process across St. Kitts farms should be reevaluated, especially when reviewing SE seropositivity, as an unwashed, unrefrigerated approach, and elimination of soiled eggs, may be more practical in the current conditions.

While seroprevalence results and differences between farms were difficult to interpret due to unknown factors, (i.e., bird age, vaccination, maternal antibodies, and / or immunosuppression), one farm in this study (farm 9) had non-vaccinated, locally sourced pullets placed in a new facility that served for comparison. Notable differences in this farm compared to others included the lack of IBV antibodies and the fewest NDV and SE antibody positive birds from other farms. Post-hoc analysis reflects this, as farm 9 had significantly lower seroprevalence between multiple farms for NDV, IBV, and SE. This might be due to the lack of IBV and NDV vaccination at day of age and a lower environmental Salmonella load, reducing the bird exposure. These results underscore the importance of biosecurity, cleaning, and disinfection and the role of vaccination in the flock epidemiology.

Imported chicks received a live B1-type B1 strain NDV vaccine at day-of-age. Lentogenic NDV strains, particularly B1 or VG/GA, are highly attenuated and are typically used as primary vaccines in a program (Martiny et al. 2025). A single live vaccine is unlikely to establish long-term antibody titers needed in layers, but can generate rolling reactions with poor application (OIE-World Organization for Animal Health 2021). Previous research on island showed a 31% NDV seroprevalence (Bolfa et al. 2019) in feral chickens, in contrast to our 62.7% that reflects the day of age vaccination effect. Other exposure opportunities could be associated with lentogenic strains carried by waterfowl or velogenic strains harbored in local chickens. Unfortunately, we cannot understand this further as we were unable to molecularly detect and characterize this virus from the collected swabs.

Imported chicks were also vaccinated with a single modified-live IBV vaccine (Mass + Conn) at the hatchery. This vaccine, when poorly applied, can induce rolling reactions throughout the flock and neighboring farms (Jackwood and Jordan 2021; Abozeid 2023). St. Kitts feral chickens had a comparable IBV seropositivity rate at 84% (Bolfa et al. 2019). Although we did not pursue genotyping, we can speculate that vaccinated chicks are the source of IBV exposure on island. This is further suggested as the only locally sourced, non-vaccinated farm 9 lacked IBV seropositive birds.

AI antibodies, while in low numbers, were detected in most farms. This is surprising in comparison to the data obtained by Bolfa (Bolfa et al. 2019). It is important to consider that the HPAI ecology has changed over the years and HPAI exposure in the island might have increased due to this (Harvey et al. 2023). The negative NDV and AIV antigen detection by RT-qPCRs in our study is most likely attributable to prior exposure and subsequent viral clearance before sample collection. Despite the inability to detect and pathotype NDV nor AIV, mass mortality events to suggest highly pathogenic strains have not been reported by farmers. There is however, inherent concern for AIV in the country, as St. Kitts is located within the Western North Atlantic migratory bird route (Rappole et al. 2000) and the Cayman Islands had an HPAI H5N1 outbreak (OIE-WAHIS 2026). These factors and the regulatory concern for this disease (OIE-World Organization for Animal Health 2025) highlights the importance of AIV surveillance and biosecurity in the region.

All commercial farms had SE antibody positive birds, compared to feral chickens with no SE antibody detections (Bolfa et al. 2019). This is not surprising as commercial birds are confined in facilities with multiple bird ages and lacking proper cleaning and disinfection programs. Suboptimal management practices (i.e., high stocking densities, few nest boxes, soil floors, and dust) can contribute to environmental SE contamination and perpetuation in multi-age flocks (Neelawala et al. 2024; Dutra et al. 2025). Most farms had scarce nest availability that was remarkably under the recommended 1 nest for every 4 hens (Lohmann Breeders 2022). Insufficient nest-to-hen ratios are known to increase the number of floor eggs and consequently fecal contamination (Dutra et al. 2025). Farm 9 had fewer seropositive birds from farms 7, 8, and 9. This was expected as birds were raised in a brand-new “clean” facility and less risk of environmental contamination. In the Caribbean, specifically in the Cayman island, a study linked imported chicks with *Salmonella* introduction (Watler et al. 2024). *Salmonella* horizontal transmission was more common than vertical transmission in Trinidad and Tobago commercial poultry (Adesiyun et al. 2014). The SE seropositivity rates among sampled farms, husbandry practices, and egg sanitation methods raise concerns for potential food safety risk to St. Kitts consumers (Donna Morrison et al. 2022) and emphasize the importance of addressing these issues.

These results underscore the critical need for ongoing surveillance to accurately characterize disease challenges and to implement effective preventive and corrective measures. Surveillance programs for *Salmonella* in the environment and eggs, while standard in most countries (Cota et al. 2024), have yet to be established on island. The presence of non-typhoidal *Salmonella* spp. pathogens in St. Kitts poultry and egg products should be investigated. Mitigation efforts (i.e., *Salmonella* surveillance programs, vaccination, and holistic approaches that consider vermin control, feed ingredient quality control, etc.) should additionally be explored. While the antibody detections of NDV and AIV indicate previous exposure and a humoral immune response, it cannot be directly linked with clinical cases. Surveillance is needed to accurately characterize these disease challenges and to implement effective preventive and corrective measures. Since our study, St. Kitts has established a layer hatchery on island to supply local farms (St. Kitts Nevis Information Service 2024b). The impact of this action in local poultry production is unknown.

It must be reinforced that the commercially available ELISA test kits used in this study do not necessarily imply viral circulation, rather demonstrating prior wild type or vaccine virus exposure. Without viral and bacterial identification, it is impossible to confirm the presence of these pathogens and further characterize them. Nevertheless, our study highlights multiple challenges within St. Kitts poultry production. A holistic approach is needed to establish simple disease prevention and food safety strategies, to ensure safe and sustainable poultry products, especially in countries under development.

## Supplementary Information

Below is the link to the electronic supplementary material.

Supplementary Material 1

## Data Availability

The data that supports the findings of this study are available upon reasonable request.

## Abbreviations

AIV: Avian influenza virus
NDV: Newcastle disease virus
IBV: Infectious bronchitis virus
SE: Salmonella enterica subspecies enterica serovar Enteritidis
HPAI: Highly pathogenic avian influenza
LPAI: Low pathogenic avian influenza
FTA: Finders Technology Associates
RT-qPCR: Reverse transcriptase quantitative polymerase chain reaction
ELISA: Enzyme-linked immunosorbent assay
