# Supplementary material for: Biosecurity assessment and seroprevalence of relevant poultry diseases in Saint Kitts commercial poultry premises
Source: Trop Anim Health Prod. 2026 May 28;58(5):287. doi: 10.1007/s11250-026-05099-2 (PMC13219060; doi:10.1007/s11250-026-05099-2)
Supplement: Supplementary file 1 — Supplementary Material 1 [file 11250_2026_5099_MOESM1_ESM.doc]

**SK Poultry Farm Survey**

Survey number Date

Farmer Name Tel.

Address (Parrish)

Location/Farm name:

How close is the nearest public road to your farm?

<50 yards

50-100 yards

>100 yards

How close is the nearest commercial poultry facility?

>1 mile

400 yards to 1 mile

<400 yards

I don't know

How many of your neighbors have backyard flocks?

0

1-5

>5

Unknown

How close is the nearest backyard flock?

Next door

Within the same neighborhood

No one else has a backyard flock near me

Unknown

How close is the nearest body of water?

On property

50- 100 yards

>100 yards

Do you see waterfowl on your property?

Commonly

Rarely

Never

Do you see non-waterfowl avian species on your property?

Commonly

Rarely

Never

**Poultry housing**

Do you have a fenced yard that helps keep your birds inside your property?

Yes

Partial fence

No

Production system

Caged

Non-caged

Access to outdoors

What type of housing do your birds live in?

Completely enclosed and no wild animals can enter

Birds have an enclosed house but sometimes escape or wild animals enter

Birds are kept inside for the night but roam the yard and have contact with wild animals

Do you use an entry and exit footbath?

Footbath is used when entering and exiting the coop

There is no footbath

Do you have clothing and footwear exclusively used when working with your flock?

We have clothes that are only used near the birds

Normal clothes are worn in and around the birds

What type of watering system do you use?

Drinkers and watering system are protected from wild bird and vermin access

Drinkers and watering system are not protected from wild bird and vermin access

How do you handle spilled feed?

Feed storage containers covered, and feed spills are cleaned up immediately and not fed to poultry

Feed storage containers and delivery systems covered but spills not immediately cleaned up but not fed to poultry

Feed storage containers and delivery systems not covered, and spills not immediately cleaned up, or spilled feed fed to poultry

Do you share equipment with your neighbors or friends?

Yes

No

Do you allow people to visit your flock?

No visitors

Only visitors who have not been around poultry

Anyone can visit the flock

**Pets, Wildlife, and Domestic Animals**

Can wild birds intermingle with your birds?

Building design restricts wild bird access and there is no evidence of wild birds

Occasional sightings of wild birds flying through poultry houses; some areas where birds can sneak in

Building design allows open access to wild birds and these are often found nesting inside poultry houses or along perches on immediate exterior of houses

Do you have wild bird feeders on your property?

Yes

No

Have you observed rodents in or around your farm?

Never seen rodents

Rodents are sporadically seen

Rodents are present

Do other animals have access to the area with the birds?

Dogs, cats, livestock, or wildlife are restricted from the premises

Dogs, cats, livestock, or wildlife are restricted from the poultry coop

Dogs, cats, livestock, or wildlife seen in poultry houses or transiting between the premises and nearby farms or seen feeding on dead birds on the premises

**Cleaning and Disinfecting**

How often do you clean your coop?

Weekly

Monthly

Semi-annually

Once a year or never

How do you clean your coop?

Every equipment is cleaned, litter and first inches of soil are removed

Only the equipment is cleaned

Only the litter is removed

We do not clean

Other:

Do you use disinfectants after cleaning your equipment and/or coop?

Yes

No

If you use disinfectants which one you use?

**Flock health & biosecurity**

Do you use a laboratory or veterinarian to help monitor your flock's health?

Yes

No

Are you familiar with the Ministry of Agriculture of Saint Kitts?

Yes

No

Do you use a veterinarian or laboratory to diagnose causes of mortality?

Yes

No

How often do you monitor your flock?

Daily

Sporadically

Never

Do you keep written records of your flock?

Daily egg production, feed consumption and mortality

Weekly egg production, feed consumption, and mortality

No, but I know how much they produce

No

Other:

What is the source of your birds?

Barbados

Miami

Other:

How do you dispose of deceased birds?

:

Do you isolate new birds from the rest of your flock?

Yes

No

Are birds transported off sites to locations with other birds and then returned? (Examples include poultry shows)

Yes, and then returned to the flock right away

Yes, and then isolated from the flock for at least a week

No

Do you have a biosecurity protocol?

Yes

No

**Farm details**

Total farm capacity (laying hens)

(______________) # of hens

Total egg production per day?

(_______________) Unit type (___________) eggs, cartons, etc.

Where do you buy your feed from

Government (Ministry of Agriculture)

Shops

Direct import

Own produced feed

Are you planning on growing the size of the farm

Yes

No

Are you planning on increasing the number of laying hens

Yes

No

What are the main challenges observed in the farm?

:

Do you keep records and how

:

What do you do with the bedding litter after you finished the production cycle?

:

**Value chain**

How do you check the current egg market price?

:

Who do you sell your eggs to?

Egg dealers (%)

Retailers (%)

Consumers (%)

What is your farm gate price (ECD)

Dozen (1 egg):

Tray (30 eggs):

Carton (210 eggs):

What is your selling price per carton if you sell to:

Egg dealers

Retailers

Consumers

What type of problems do you encounter in the SK egg market?

:

What type of egg marketing intervention should be required from the government?

:

Do you mark your eggs

Yes

No

Do you label/brand your boxes

Yes

No

Where do you get veterinary supplies from?

:

Where do you get vitamins, calcium, supplements from?

Open

What do you do with the old hens?

Sold on site to consumers

Eliminate

Sold to other farms

Given to people

**Survey #**

**Egg food safety**

How many nests do you have

# Hens ( ) # nests ( )

# Hens ( ) # nests ( )

# Hens ( ) # nests ( )

# Hens ( ) # nests ( )

Do you wash your eggs

Yes

Yes, only the dirty ones

No, never

How do you wash your eggs

Do you use disinfectant or product for the water to clean eggs

Yes (which one)

No

Do you use water to clean your eggs

A bowl of water

Running water

With what do you wipe your eggs

Fabric cloth

Paper cloth

Other

How often do you change the cloth/towel

After each egg

After a while

Daily

Other
